# Enhancement of Osteoblast Function through Extracellular Vesicles Derived from Adipose-Derived Stem Cells

**DOI:** 10.3390/biomedicines10071752

**Published:** 2022-07-21

**Authors:** Mei-Ling Ho, Chin-Jung Hsu, Che-Wei Wu, Ling-Hua Chang, Jhen-Wei Chen, Chung-Hwan Chen, Kui-Chou Huang, Je-Ken Chang, Shun-Cheng Wu, Pei-Lin Shao

**Affiliations:** 1 Orthopaedic Research Center, College of Medicine, Kaohsiung Medical University, Kaohsiung 80787, Taiwan; homelin@kmu.edu.tw (M.-L.H.); tkdiven@gmail.com (C.-W.W.); linghua_chang@yahoo.com.tw (L.-H.C.); h90085663@gmail.com (J.-W.C.); hwan@kmu.edu.tw (C.-H.C.); jkchang@kmu.edu.tw (J.-K.C.); 2 Regenerative Medicine and Cell Therapy Research Center, Kaohsiung Medical University, Kaohsiung 80787, Taiwan; 3 Department of Physiology, College of Medicine, Kaohsiung Medical University, Kaohsiung 80787, Taiwan; 4 Graduate Institute of Medicine, College of Medicine, Kaohsiung Medical University, Kaohsiung 80787, Taiwan; 5 Department of Marine Biotechnology and Resources, National Sun Yat-Sen University, Kaohsiung 804201, Taiwan; 6 Department of Medical Research, Kaohsiung Medical University Hospital, Kaohsiung 80756, Taiwan; 7 Department of Orthopedics, China Medical University Hospital, Taichung 404332, Taiwan; jeffrey59835983@gmail.com; 8 School of Chinese Medicine, China Medical University, Taichung 406040, Taiwan; 9 Department of Orthopedics, Kaohsiung Medical University Hospital, Kaohsiung Medical University, Kaohsiung 80787, Taiwan; 10 Department of Orthopedics, College of Medicine, Kaohsiung Medical University, Kaohsiung 80787, Taiwan; 11 Department of Orthopedics, Kaohsiung Municipal Ta-Tung Hospital, Kaohsiung Medical University, Kaohsiung 80787, Taiwan; 12 Division of Adult Reconstruction Surgery, Department of Orthopedics, Kaohsiung Medical University Hospital, Kaohsiung Medical University, Kaohsiung 80787, Taiwan; 13 Program in Biomedical Engineering, College of Medicine, Kaohsiung Medical University, Kaohsiung 80708, Taiwan; 14 Department of Orthopedics, Asia University Hospital, Taichung 413505, Taiwan; kuichouhuang@gmail.com; 15 Department of Occupational Therapy, Asia University, Taichung 41354, Taiwan; 16 Post-Baccalaureate Program in Nursing, Asia University, Taichung 41354, Taiwan; 17 Department of Nursing, Asia University, Taichung 41354, Taiwan

**Keywords:** bone tissue engineering (BTE), adipose-derived stem cells (ADSCs), extracellular vesicles (EVs), osteoblast function, microRNA (miRNA)

## Abstract

Adipose-derived stem cells (ADSCs) are a type of mesenchymal stem cell that is investigated in bone tissue engineering (BTE). Osteoblasts are the main cells responsible for bone formation in vivo and directing ADSCs to form osteoblasts through osteogenesis is a research topic in BTE. In addition to the osteogenesis of ADSCs into osteoblasts, the crosstalk of ADSCs with osteoblasts through the secretion of extracellular vesicles (EVs) may also contribute to bone formation in ADSC-based BTE. We investigated the effect of ADSC-secreted EVs (ADSC-EVs) on osteoblast function. ADSC-EVs (size ≤ 1000 nm) were isolated from the culture supernatant of ADSCs through ultracentrifugation. The ADSC-EVs were observed to be spherical under a transmission electron microscope. The ADSC-EVs were positive for CD9, CD81, and Alix, but β-actin was not detected. ADSC-EV treatment did not change survival but did increase osteoblast proliferation and activity. The 48 most abundant known microRNAs (miRNAs) identified within the ADSC-EVs were selected and then subjected to gene ontology (GO) and Kyoto Encyclopedia of Genes and Genomes (KEGG) analyses. The GO analysis revealed that these miRNAs are highly relevant to skeletal system morphogenesis and bone development. The KEGG analysis indicated that these miRNAs may regulate osteoblast function through autophagy or the mitogen-activated protein kinase or Ras-related protein 1 signaling pathway. These results suggest that ADSC-EVs enhance osteoblast function and can contribute to bone regeneration in ADSC-based BTE.

## 1. Introduction

Bone fracture is the most common traumatic injury in *humans* [1]. Although the bone normally heals itself, approximately 10% of bone fractures do not heal normally [2,3]. Aging and disease are factors that can negatively affect bone healing [3]. Moreover, large bone defects due to heavy trauma or multiple fracture also disrupt normal bone healing [4,5]. Bone grafting is the current gold standard for bone defect reconstruction; it involves transplanting autologous bone tissue into the bone defect zone to trigger bone regeneration [1,3]. However, the limitations of bone grafting include donor site morbidity and potentially low availability of a suitable autologous material [1,3]. The regeneration of bone defects remains an unmet clinical need. To overcome the limitations of bone grafting, bone tissue engineering (BTE) was proposed as a promising alternative [6].

BTE relies on three key elements: (1) cells, (2) a scaffold, and (3) bioactive factors [7]. In BTE, mesenchymal stem cells (MSCs) can be a cell source because of their ability to form osteoblasts through osteogenic differentiation [7]. MSCs derived from adipose tissue (adipose-derived stem cells: ADSCs) or bone marrow (bone marrow–derived stem cells: BMSCs) are the MSCs most commonly employed in BTE [7,8]. In contrast to BMSCs, ADSCs are an attractive cell source for MSC-based BTE because of the straightforward noninvasive methods used to harvest them, their high cell yield, and their high proliferation capacity during in vitro culture [7]. Studies have explored the application of ADSCs in BTE in both preclinical and clinical settings [3,9,10].

A common BTE strategy is to implant MSCs in combination with active molecules to induce the osteogenic differentiation of the MSCs into osteoblasts to enhance new bone formation [3,11]. Within a bone defect zone, MSCs undergo osteogenic differentiation and then form bone [3]. Researchers initially assumed that the osteogenic differentiation potential of MSCs is crucial in BTE. However, studies have reported the poor differentiation of MSCs at injury sites and the lack of a correlation between the osteogenic differentiation potential and favorable clinical results [3,12,13]. In addition to performing the osteogenic differentiation of MSCs in situ, scholars have demonstrated that the cell-to-cell crosstalk caused by the secretome of MSCs [14,15] also contributes to enhanced bone regeneration in BTE [3,16]. The secretome comprises extracellular vesicles (EVs), peptides, and small proteins (cytokines) [17,18]. MSCs are an abundant source of EVs [19]. Studies have indicated that ADSC-secreted EVs (ADSC-EVs) promote bone regeneration by providing benefits such as anti-inflammation, angiogenesis, and osteoinduction [10,20,21]. Osteoblasts are the main cells responsible for bone formation during bone regeneration [22], and their functions (e.g., survival, proliferation, and activity) play key roles in bone formation and repair [23,24,25]. However, only few studies focus on the effect of MSC-EVs on osteogenic differentiation of the MSCs into osteoblasts [20,26,27]. Several studies tested the effect of MSCs-EVs on osteoblasts in the diseases such as osteoarthritis or osteoporosis [28,29,30]. For example, a previous study reported that ADSC-EVs downregulate senescence features in osteoarthritic osteoblasts [28]. For ADSC-based BTE, whether ADSC-EVs promote osteoblast function remains rarely investigated.

In the present study, we hypothesized that ADSC-EVs promote osteoblast function. To test our hypothesis, we isolated and characterized ADSC-EVs and tested their effect on osteoblast function (survival, proliferation, and activity). We also analyzed the microRNA (miRNA) profile of ADSC-EVs to clarify their possible molecular mechanism.

## 2. Materials and Methods

### 2.1. Materials

All chemicals were purchased from Sigma-Aldrich (Sigma-Aldrich, St. Louis, MO, USA) unless otherwise specified.

### 2.2. Human ADSCs

The *human* ADSCs used in the present study were purchased from StemPro (StemPro *Human* Adipose-Derived Stem Cells, Gibco BRL, Thermo Fisher Scientific, Waltham, MA, USA). *Human* ADSCs were cultured in K-NAC medium for ADSC expansion per the protocol presented in our previous papers [31,32]. The K-NAC medium comprised a keratinocyte SFM basal medium (Gibco BRL, Rockville, MD, USA) supplemented with 25 mg of bovine pituitary extract (Gibco BRL), 2.5 µg of *human* recombinant epidermal growth factor (Gibco BRL), 2-mM N-acetyl-L-cysteine, 0.2-mM L-ascorbic acid 2-phosphate sesquimagnesium salt, and 5% fetal bovine serum (FBS) [31,32]. The cells were cultured and expanded in a humidified atmosphere with 5% CO_2_ at 37 °C until a sufficient number of cells was acquired.

### 2.3. Human Osteoblasts

The *human* nontumor osteoblast (hFOB) cell line (hFOB 1.19, ATCC CRL-1137, Manassas, VA, USA) [33] and primary normal *human* osteoblasts (hOBs; Clonetic; Lonza, Basel, Switzerland) were used in the present study. To expand the hFOBs, cells were cultured and expanded in a 1:1 mixture of Ham’s F12 medium and Dulbecco’s modified Eagle’s medium (DMEM) containing 2.5-mM L-glutamine supplemented with 100-U/mL penicillin/streptomycin (Gibco BRL, Thermo Fisher Scientific, Waltham, MA, USA), 0.3-mg/mL G418, and 10% FBS [33]. The hFOBs were expanded at 34 °C in an atmosphere with 5% CO_2_, and the medium was changed every 2 days until a sufficient number of cells was detected [34]. To expand the hOBs, the cells were cultured and expanded in DMEM containing 100-mg/mL ascorbic acid, nonessential amino acids, penicillin/streptomycin, and 10% FBS [35]. The hOBs were maintained in a humidified atmosphere containing 5% CO_2_ at 37 °C. For all experiments, the hFOBs and hOBs were used within five to six subcultures.

### 2.4. Induction and Isolation of ADSC-EVs

For the induction of ADSC-EVs, ADSCs were suspended within DMEM supplemented with 10% EV-depleted FBS (exosome-depleted, One Shot^TM^ format; Catalog number, A2720803; Gibco BRL) and 100-U/mL penicillin/streptomycin (Gibco BRL). The ADSCs were then seeded on 15-cm culture plates (1.5 × 10^4^ cells/cm^2^) and cultured at 37 °C for 48 h [36,37]. After 48 h, the conditioned medium (CM) in which the ADSC-EVs had been secreted was collected. A multistep ultracentrifugation protocol was then applied to isolate the ADSC-EVs with minimal modification [36,37,38]. To remove the suspended ADSCs within the CM, the CM was centrifuged at 2000× *g* for 10 min at 4 °C, and the supernatant was collected for further centrifugation. The collected supernatant was centrifuged at 20,000× *g* for 10 min at 4 °C to remove large cell fragments [38]. The supernatant was then centrifuged at 50,000× *g* for 1 h to remove residual organelles and nuclei [38]. Subsequently, it was centrifuged at 180,000× *g* for 1.5 h at 4 °C to pellet the ADSC-EVs [38]. Thereafter, the supernatant was discarded, and the ADSC-EVs at the bottom of the centrifuge tube were resuspended in phosphate-buffered saline (PBS) to eliminate the contaminating proteins on the ADSC-EVs pellets and centrifuged at 180,000× *g* for 1.5 h at 4 °C to acquire ADSC-EVs pellets [38]. The ADSC-EVs were resuspended in 1 mL of sterilized PBS and stored at 4 °C until characterization or treatment [36]. The characterization of the ADSC-EVs involved analyses of their size, number, morphology, and protein composition [36,39]. The isolated ADSC-EVs stored at 4 °C were used within 2 weeks.

### 2.5. Nanoparticle Tracking Analysis of ADSC-EV Size and Number

Immediately after the ADSC-EVs was isolated, their particle size distribution and particle number were measured using NanoSight (LM10; Malvern, UK) [36]. First, the ADSC-EVs were suspended in 1 mL of PBS, which was filtered using a 0.22-μm filter (Pall Corporation, New York, NY, USA). Before the ADSC-EV suspension was loaded into a sample chamber for visualization using the NanoSight LM10, it was diluted 2000-fold in PBS to obtain a concentration within the recommended measurement range (20–100 particles/frame). The software settings for this analysis were as follows: temperature = 20–23 °C, and number of frames = 3. The particle size distribution and particle number of the ADSC-EVs were then measured.

### 2.6. Transmission Electron Microscopy Analysis of ADSC-EV Morphology

The morphology of the ADSC-EVs was analyzed through transmission electron microscopy (TEM) [36]. Freshly isolated ADSC-EVs were suspended in distilled water. A 10-μL ADSC-EV sample was placed on a carbon-coated 200-mesh copper grid (Agar Scientific, Essex, UK) and dried at 37 °C. The sample was stained with 1% phosphotungstic acid (Scharlau, Barcelona, Spain) and then dried at 37 °C overnight. The grids were viewed through TEM (JEM-1400 Plus; JEOL, Tokyo, Japan) at an acceleration voltage of 80 kV, and digital images were captured.

### 2.7. Western Blot Analysis of ADSC-EV Protein Composition

The protein composition of the ADSC-EVs was analyzed through Western blotting [36]. The ADSC-EVs that were freshly isolated after ultracentrifugation were immediately lysed using a radioimmunoprecipitation assay buffer (Sigma, St. Louis, MO, USA) containing a protease inhibitor cocktail (Biotools, New Taipei City, Taiwan). The lysate was separated using 12% sodium dodecyl sulfate–polyacrylamide gel electrophoresis (100 V, 2 h) and transferred onto a polyvinylidene difluoride (PVDF) membrane (Bio-Rad Laboratories, Hercules, CA, USA). The PVDF membrane was blocked at room temperature for 1 h with 5% bovine serum albumin in PBS containing 0.1% Tween-20 (PBS-T). Subsequently, the membrane was incubated at 4°C overnight with primary antibodies against *CD9* (category no. 60232-1-lg; Proteintech Group; 1:1000), CD81 (category no. 66866-1-lg; Proteintech Group; 1:1000), Alix (Proteintech Group; category no. 12422-1-AP; 1:1000), and β-actin (category no. 66009-1-lg; Proteintech Group; 1:1000). After the membranes were washed with PBS-T three times, they were incubated with horseradish peroxidase (HRP)-conjugated secondary antibodies (goat anti-mouse IgG (category no. C04001-100) or goat anti-rabbit IgG (category no. C04003-100); Proteintech Group; 1:5000) at room temperature for 2–4 h. Bands were detected using a chemiluminescence kit (SuperSignal West Pico PLUS Chemiluminescent Substrate; Thermo Fisher Scientific). Chemiluminescence signals were captured using a FUSION-FX imaging system (Vilber Lourmat, Collégien, France).

### 2.8. ADSC-EV Treatment of Osteoblasts

The hFOBs and hOBs were first seeded onto six-well plates at a concentration of 10^5^ cells/well. After 24 h, the hFOBs and hOBs were treated with the ADSC-EVs. For the ADSC-EV treatment, the ADSC-EV solution was diluted with osteoinduction medium immediately before the start of treatment. The ADSC-EV concentrations used in the experiments were between 10^7^ and 10^9^ particles/mL. The osteoinduction medium comprised DMEM supplemented with L-ascorbic acid-2-phosphate (50 μM), β-glycerophosphate disodium (10 mM), and dexamethasone (0.1 μM) [32,40]. The hFOBs and hOBs were treated with ADSC-EVs (10^7^–10^9^ particles/mL) for 12 days. Two groups were examined in the present study, namely the (1) Control: control group in which the hFOBs and hOBs were cultured in the osteoinduction medium without ADSC-EV treatment and (2) EVs: EV group in which the hFOBs and hOBs were cultured in the osteoinduction medium and treated with ADSC-EVs at concentrations of 10^7^–10^9^ particles/mL. The medium was changed every 2 days. At specific time points, the hFOBs and hOBs were collected for further experimental analysis.

### 2.9. Labeling of ADSC-EVs and Uptake by Osteoblasts

For the labeling of ADSC-EVs, the ADSCs were first labeled with CellTracker^TM^ CM-DiI Dye (Sigma, St. Louis, MO, USA) per the manufacturer’s protocol. Subsequently, the ADSC-EVs were induced and isolated using the protocol described in an earlier subsection, and the CM-DiI-labeled ADSC-EVs were isolated through ultracentrifugation. For the uptake of ADSC-EVs by osteoblasts, the hFOBs (10^4^ cells/well) and hOBs (10^5^ cells/well) were seeded onto six-well plates. After 24 h, the hFOBs and hOBs were cultured for 5 days with osteoinduction medium containing 10^9^ particles/mL of CM-DiI-labeled ADSC-EVs. The medium was changed every 2 days. At specific time points, the cells were fixed with 4% paraformaldehyde in PBS for 15 min. The hFOBs and hOBs were stained with CellTracker™ Green CMFDA Dye (Invitrogen, Carlsbad, CA, USA) for cell tracing, and their cell nuclei were stained with 4′,6-diamidino-2-phenylindole. The uptake of the CM-DiI-labeled ADSC-EVs by the osteoblasts was visualized through confocal laser scanning microscopy (Zeiss, Weimar, Germany). Images were captured using a camera.

### 2.10. Cell Survival of Osteoblasts after ADSC-EV Treatment

The survival of the osteoblasts after ADSC-EV treatment was assessed using a Dr. View live/dead dual-staining kit for mammalian cells (IMT FORMOSA New Materials, Kaohsiung, Taiwan). Live/dead images of the hFOBs and hOBs were captured at days 1 and 5 after the ADSC-EV treatment. The medium was discarded, and the hFOBs and hOBs were washed twice with PBS. Cell survival was assessed on the basis of the integrity of the cellular membrane by using the aforementioned Dr. View live/dead dual-staining kit, which contains calcein-AM (live cell dye, green) and ethidium homodimer-1 (EthD-1; dead cell dye, red). A dye solution was made by combining 0.5 μL of calcein-AM with 2 μL of EthD-1 in 1 mL of osteoinduction medium. The hFOBs and hOBs were incubated in 1 mL of the live/dead dye solution in each well for 15 min. The live/dead dye solution was then removed, and the hFOBs and hOBs were viewed under a fluorescence microscope with 494-nm (green, calcein) and 528-nm (red, EthD-1) excitation filters (Zeiss ApoTome, Oberkochen, Germany).

### 2.11. Cell Proliferation of Osteoblasts after ADSC-EV Treatment

A CellTiter 96^®^ AQueous One Solution Cell Proliferation Assay (Promega, Madison, WI, USA) was used to count cells; this assay is a colorimetric method for determining the number of viable cells in a culture [41]. The mitochondrial activity of the hFOBs and hOBs cultured on wells was detected through the conversion of 3-(4,5-dimethylthiazol-2-yl)-2,5-diphenyltetrazolium bromide (MTS) into formazan by following the protocol employed in other studies [41,42,43], and the quantity of formazan product released into the medium, which is directly proportional to the number of living cells in the culture, was measured through absorbance at 490 nm [41]. At specific time points, a freshly prepared MTS reaction mixture diluted in osteoinduction medium at a 1:5 (MTS:medium) volume ratio was added to the wells containing the hFOBs and hOBs and then incubated for an additional 4 h at 37 °C in an atmosphere with 5% CO_2_. After the additional incubation, 100 μL of the converted MTS released into the medium from each well was transferred to a 96-well plate, and the absorbance at 490 nm was recorded with a microplate reader (PathTech) by using KC junior software.

### 2.12. RNA Isolation and Quantitative Real-Time Polymerase Chain Reaction (qRT-PCR)

At the indicated time points, the hFOBs and hOBs were collected. The TOOLSmart RNA Extractor (Biotools, New Taipei City, Taiwan) was used to extract the total RNA from these cells. The RNA’s quality was confirmed by determining the ratio of absorbance at 260 versus 280 nm by using a Thermo Scientific NanoDrop^TM^ 1000 spectrophotometer (Thermo Fisher Scientific, Waltham, MA, USA). Per the manufacturer’s instructions, a 260/280-nm absorbance ratio of between 1.8 and 2.0 indicated the absence of DNA contamination. Subsequently, 0.5–1 μg of total RNA per 20 μL of reaction volume was reverse-transcribed into cDNA by using the TOOLS Easy Fast RT Kit (Biotools, New Taipei City, Taiwan). Real-time PCR reactions were performed and monitored using TOOLS 2X SYBR qPCR Mix (Biotools, New Taipei City, Taiwan) and a qRT PCR detection system (Bio-Rad Laboratories, Hercules, CA, USA). The cDNA samples (2-µL samples with a total volume of 25 µL per reaction) were analyzed for the genes of interest. The mRNA levels of runt-related transcription factor 2 (*Runx2*), collagen type I (*Col-I*), alkaline phosphatase (*ALP*), osteocalcin (*OC*), and glyceraldehyde-3-phosphate-dehydrogenase (*GAPDH*) were quantified using the following PCR primer pairs: *Runx2* (forward: ACA GCT GGG GAC ATT AGT GG; reverse: GTG GAA TGC AGA GGT GGT TT); *Col-I* (forward: GGC TCC TGC TCC TCT TAG; reverse: CAG TTC TTG GTC TCG TCA C); *ALP* (forward: CCT CCT CGG AAG ACA CTC TG; reverse: GCA GTG AAG GGC TTC TTG TC); *OC* (forward: GTG CAG AGT CCA GCA AAG GT; reverse: CGA TAG GCC TCC TGA AAG C); and *GAPDH* (forward: TCT CCT CTG ACT TCA ACA GCG AC; reverse: CCC TGT TGC TGT AGC CAA ATT C). The following cycling conditions were applied: incubation at 94 °C for 1 min, followed by 35 cycles of denaturation at 94 °C for 30 s and annealing and extension at 59 °C for 30 s. After the real-time PCR reaction, a dissociation (melting) curve was generated to determine the specificity of the reaction. The relative mRNA expression level of each target gene was calculated from the threshold cycle (Ct) value of each PCR product and normalized to *GAPDH* expression by using the comparative Ct method [44]. For each gene of interest, the readings of four wells from each experimental group were collected at every examined time point.

### 2.13. Von Kossa Staining of Calcium Deposition by Osteoblasts after ADSC-EV Treatment

Calcium that was deposited in the calcified matrix by osteoblasts was visualized using von Kossa staining [45]. At specific time points, the hFOBs and hOBs were washed twice with PBS and fixed using 10% formalin for 30 min. The cells were incubated in 5% silver nitrate (Sigma-Aldrich, St. Louis, MO, USA) under a lamp for 20 min until the calcium turned black; after which, the cells were washed with 5% sodium thiosulfate for 5 min. Through the aforementioned staining method, the mineral matrix was stained black. Images were captured using a camera.

### 2.14. Alizarin Red S Staining and Quantification of Calcium Deposition by Osteoblasts after ADSC-EV Treatment

The amount of deposited calcium in the calcified matrix was measured through Alizarin red S staining and quantification [46]. At each examined time point, the hFOBs and hOBs were fixed with 0.05% (*v*/*v*) glutaraldehyde at room temperature for 10 min and then washed with distilled water. The fixed hFOBs and hOBs were then incubated with Alizarin red S (1% concentration in distilled water, pH 4.2) for 5 min and then extensively washed with distilled water. The fixed and stained plates were subsequently air-dried at room temperature. The amount of calcium mineral deposit was measured by dissolving the cell-bound Alizarin red S in 10% acetic acid and then performing spectrophotometric quantification at a wavelength of 415 nm.

### 2.15. Enzyme-Linked Immunosorbent Assay (ELISA) for Quantification of Col-I Synthesis by Osteoblasts after ADSC-EV Treatment

The amount of *Col-I* synthesized by osteoblasts was measured using a *human* collagen type I alpha 1 ELISA kit (category no. RK01149; ABclonal). The hFOBs and hOBs were incubated with 0.1% pepsin in 10% acetic acid at 55 °C for 1 h for cell lysis. Next, 100 μL of the cell lysate was placed in an antibody-coated well for 2 h at 37 °C, washed three times, and incubated with a biotin conjugate antibody for 0.5 h at 37 °C. Washing and incubation with streptavidin-HRP were then conducted for 20 min at 37 °C in the dark. The synthesis of Col-I by hFOBs and hOBs was measured at an absorbance wavelength of 570 nm by using an ELISA reader (BioTek Synergy H1).

### 2.16. ELISA for Quantification of ALP Activity by Osteoblasts after ADSC-EV Treatment

The quantification of *ALP* activity by osteoblasts was measured using a CheKine™ Tissue and Blood Alkaline Phosphatase (AKP/ALP) Activity Colorimetric Assay Kit (category no. KTB 1700). The hFOBs and hOBs were incubated with extraction buffer at 4 °C for 1 h for cell lysis and centrifuged at 10,000× *g* for 10 min at 4 °C to acquire the cell lysate. Next, 100 μL of cell lysate was mixed with 100 μL of chromogen A+ chromogen B for 15 min at 37 °C, then mixed with 200 μL of chromogen C. The *ALP* activity by hFOBs and hOBs was measured at an absorbance wavelength of 510 nm by using an ELISA reader (BioTek Synergy H1).

### 2.17. Small RNA Sequencing and KEGG and Gene Ontology (GO) Analysis of ADSC-EVs

#### 2.17.1. miRNA Extraction

The extraction of miRNA from ADSC-EVs was performed using an EVmiR Extraction Kit (Topgen Biotech., Kaohsiung, TW) per the manufacturer’s procedure, and the extracted miRNA from each sample was then eluted in 20 μL of low TE buffer. The concentration of miRNA was quantified using a Qubit microRNA Assay Kit on a Qubit Fluorometer (Thermo Fisher, USA).

#### 2.17.2. miRNA Library Construction for Next-Generation Sequencing

A total of 7 μL of the microRNA sample extracted from ADSC-EVs was used to construct a miRNA library with the VAHTS Small RNA Library Prep Kit for Illumina (Vazyme Biotech, CN, Nanjing, China) and VAHTS Small RNA Index Primer Kit for Illumina (Vazyme Biotech, CN) per the manufacturer’s instructions. The appropriate barcoding adapter-ligated library amplicon length was approximately 142 bp, and the amplicons in the target region were excised from 4% agarose gel and purified using a FastPure Gel DNA Extraction Mini Kit (Vazyme Biotech, CN). All steps were performed per the manufacturer’s instructions. Microfluidic electrophoresis was performed using the MultiNA MCE-202 DNA-2500 Kit (Shimadzu, Kyoto, Japan) to quantify the concentration and verify the length distribution of the eluted barcoding adapter-ligated library (142 bp). The qualified pooled library (4 nM) was sequenced on an Illumina NextSeq 500 (single-end, 75 sequencing cycles; San Diego, CA, USA). All steps were performed per the manufacturer’s instructions.

#### 2.17.3. Bioinformatics Analysis

First, the CLIP Tool Kit (v. 1.0.3) was used to trim the total reads to 34 bp on the 3′ end to remove the adapter sequence [47]. Subsequently, an appropriate insert size of approximately 15–30 bp for mature miRNA was retained using Filter FASTQ (v. 1.1.5) [48]. The trimmed reads were mapped with the *human* microRNA database version 22.1 (miRbase.org) by using BWA (v. 0.7.17.4) [49]. After the mapped reads of each sample were assembled and quantified using Linux commands, edgeR (v. 3.32.1) [50] was used to estimate the expression levels of all miRNAs. Differentially expressed miRNAs and genes were selected on the basis of a log2 (fold change) > 1 or log2 (fold change) < −1 at the significance level of 0.05 by using the R software package. The target genes of the expressed miRNA that was highly differentiated were predicted using Targetscan version 7.2 (targetscan.org), miRanda (www.microrna.org, accessed on 24 June 2021), and miRDB (http://www.mirdb.org/, accessed on 24 June 2021). The union of the target genes was achieved by conducting GO and Kyoto Encyclopedia of Genes and Genomes (KEGG) pathway enrichment analyses through the use of ClusterProfiler (v. 3.18.1) [51] (*p*-value < 0.05; q-value < 0.05) and ggplot2 (v. 3.3.3) [52] to visualize the enrichment plots.

### 2.18. Statistical Analysis

The data were expressed as the mean ± standard deviation (SD, i.e., SD of the mean of the combined data of the experimental replicates). Statistical significance was evaluated through *t*-tests, with a *p*-value of <0.05 indicating statistical significance.

## 3. Results

### 3.1. Characterization of ADSC-EVs

**Figure 1:**
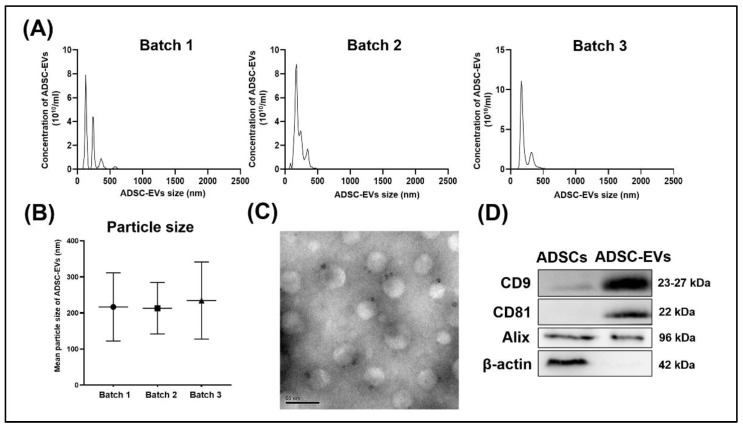
Characterization of ADSC-EVs. ADSC-EVs were isolated from three separate batches of conditioned media (CMs) and characterized. (**A**) Particle size distribution of ADSC-EVs measured through a nanoparticle tracking analysis (NTA). (**B**) Mean particle sizes of ADSC-EVs measured through NTA. Data are presented as the mean ± standard deviation (SD; *n* = 3). (**C**) Morphology of the ADSC-EVs as observed through transmission electron microscopy. (**D**) Western blot analysis of the protein levels of *CD9, CD81, Alix*, and *β-actin* in ADSCs and ADSC-EVs.

ADSC-EVs were isolated from three separate batches of ADSC-cultured CMs through ultracentrifugation. The particle size, particle number, morphology, and protein composition of the isolated ADSC-EVs were analyzed. The particle size and number of isolated ADSC-EVs were analyzed through nanoparticle tracking analysis (NTA). The NTA results revealed that the sizes of the ADSC-EVs ranged from 0 to 1000 nm (Figure 1A). The mean particle sizes of the ADSC-EVs from the three batches were 216.9 ± 94.6, 213.3 ± 71.4, and 234.5 ± 106.8 nm (Figure 1B). No significant difference in the mean particle size was calculated among the ADSC-EVs from these three batches. The morphology of the ADSC-EVs was also characterized through TEM; the captured images indicated that the ADSC-EVs were spherical (Figure 1C). The protein composition of the ADSC-EVs (determined through Western blotting) indicated that the ADSC-EVs were positive for *CD9*, *CD81*, and *Alix* but negative for β-*actin* (Figure 1D). Collectively, these results indicated that the ADSC-EVs were secreted into the CM by ADSCs and successfully isolated from the CM.

### 3.2. ADSC-EV Uptake by Osteoblasts

**Figure 2:**
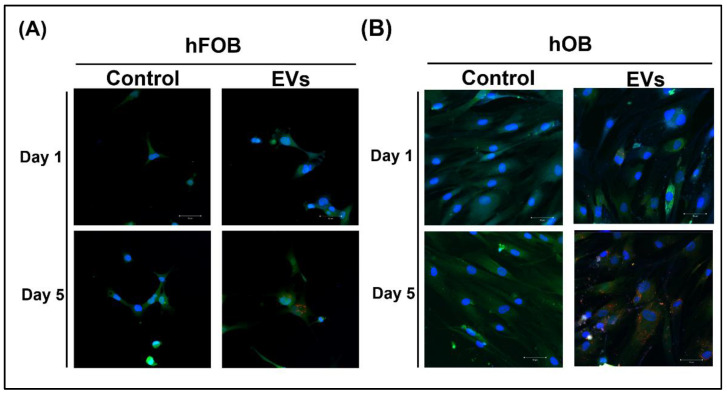
ADSC-EV uptake by osteoblasts. The hFOBs (**A**) and hOBs (**B**) were treated with CM-DiI-labeled ADSC-EVs at concentrations of 0 (Control: control group) or 1 × 10^9^ particles/mL (EVs: EV group) for 5 days, and images at days 1 and 5 were obtained using a camera under confocal microscopy. Cell nucleus, blue fluorescence stain; cytoplasm, green fluorescence stain; and CM-DiI-labeled ADSC-EVs, red fluorescence stain.

To investigate whether cell-to-cell communication between the ADSCs and osteoblasts occurred through the ADSC-EVs, CM-DiI dye was used to label the ADSC-EVs and monitor their delivery into osteoblasts. We cocultured CM-DiI-labeled ADSC-EVs with hFOBs or hOBs and observed the uptake of ADSC-EV by cells for 5 days. No CM-DiI-labeled ADSC-EVs were found within the hFOBs in either the control or EV group on day 1 (Figure 2A). The uptake of CM-DiI-labeled ADSC-EVs by hOBs was limited in the EV group on day 1 (Figure 2B). On day 5, the results for both the hFOBs and hOBs indicated that the CM-DiI-labeled ADSC-EVs were localized in the cytoplasm and around cell nuclei in the EV group; by contrast, no CM-DiI-labeled ADSC-EVs were detected in the control group (Figure 2). These results revealed the uptake of the ADSC-EVs by the hFOBs and hOBs after 5 days of incubation; they also suggested that cell-to-cell communication between ADSCs and osteoblasts occurs through the delivery of ADSC-EVs.

### 3.3. Effect of ADSC-EVs on Survival and Proliferation of Osteoblasts

**Figure 3:**
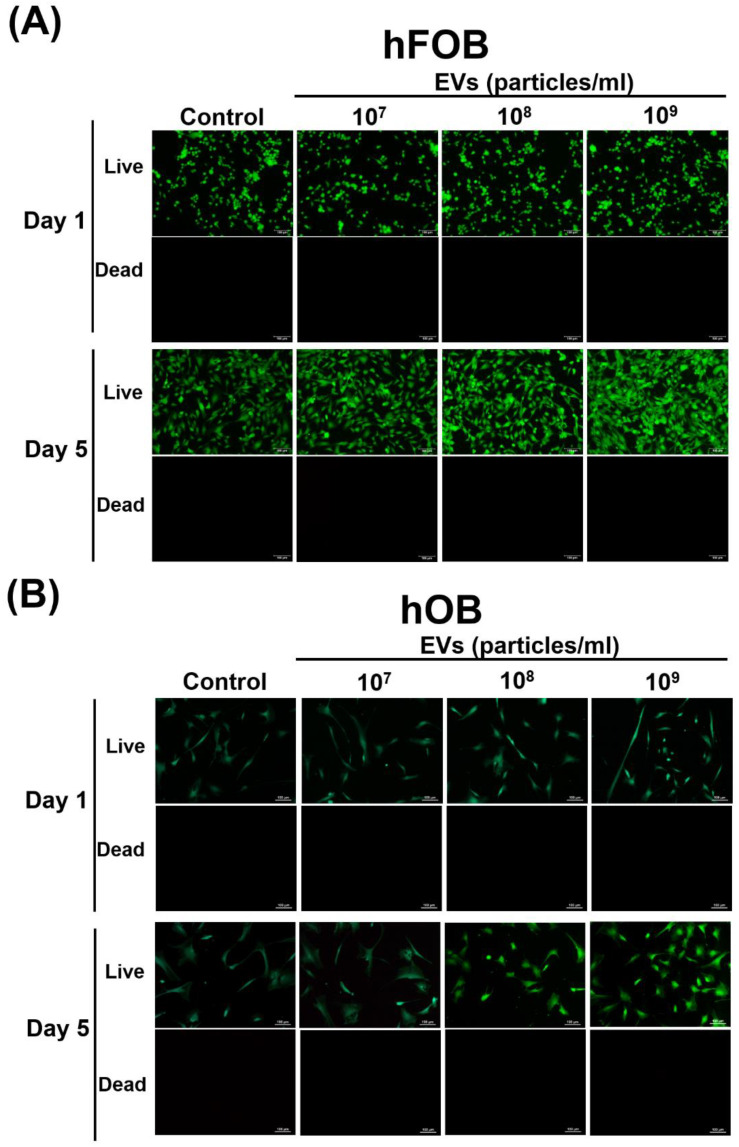
Effect of ADSC-EVs on the survival of osteoblasts. The hFOBs (**A**) and hOBs (**B**) were treated with ADSC-EVs at concentrations of 0 (Control: control group) or 10^9^ particles/mL (EVs: EV group) for 5 days and analyzed for survival. Live/dead cell assays were performed for hFOBs and hOBs to determine the cell survival on days 1 and 5. Green fluorescence indicates live cells (Live), whereas red fluorescence indicates dead cells (Dead). The hFOBs and hOBs remained alive on days 1 and 5 after the ADSC-EV treatment.

**Figure 4:**
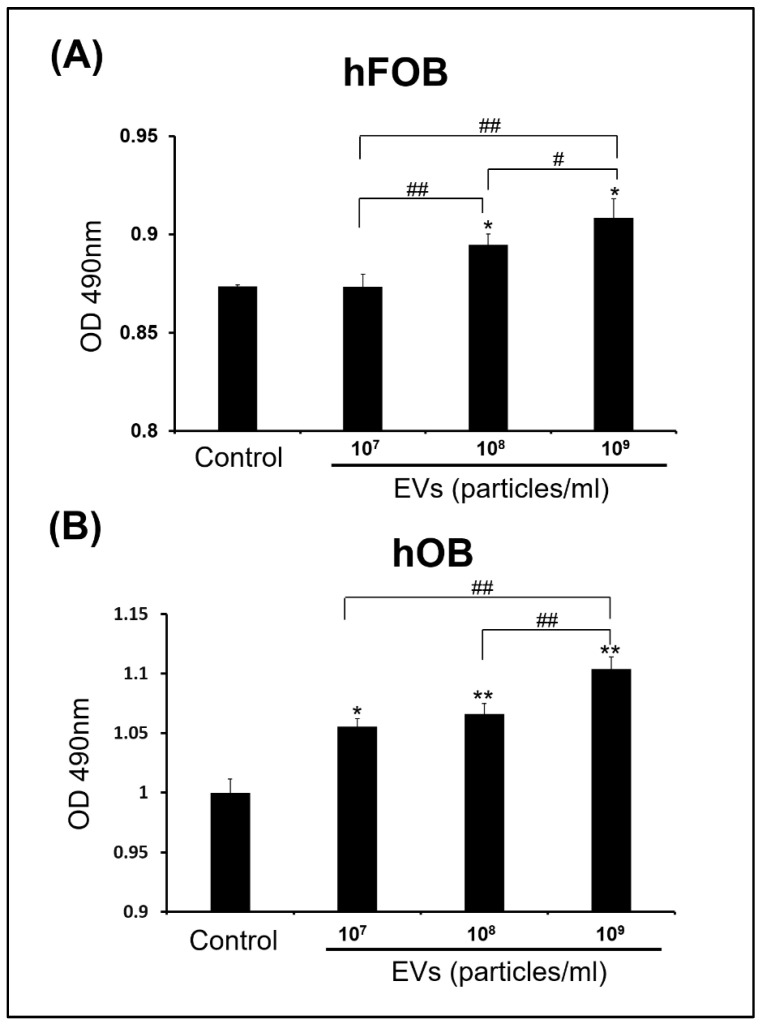
Effect of ADSC-EVs on the proliferation of osteoblasts. The hFOBs (**A**) and hOBs (**B**) were treated with ADSC-EVs at concentrations of 0 (Control: control group) or 1 × 10^7^–1 × 10^9^ particles/mL (EVs: EV group) for 5 days and analyzed for cell proliferation. MTS assays were performed for hFOBs and hOBs on day 5 to determine the cell proliferation. Cell proliferation of hFOBs and hOBs was enhanced after the ADSC-EV treatment. Data are presented as the mean ± SD (*n* = 6). * *p* < 0.05 and ** *p* < 0.01 for comparisons with the control group. # *p* < 0.05 and ## *p* < 0.01 for comparisons between the two groups.

To determine whether ADSC-EVs influence the survival and proliferation of osteoblasts, the hFOBs and hOBs were incubated with various concentrations (10^7^–10^9^ particles/mL) of ADSC-EVs for 5 days. The results indicated that the ADSC-EVs did not influence the survival of the osteoblasts. The live/dead staining revealed that the hFOBs and hOBs were alive, and no obviously dead cells were detected in either the control or EV group on days 1 and 5 (Figure 3A,B). Regarding cell proliferation, we discovered that the ADSC-EV treatment promoted the proliferation of osteoblasts. In the hFOBs, enhanced cell proliferation was detected in the EV group relative to that in the control group at the concentrations of 10^8^ and 10^9^ particles/mL (Figure 4A). A similar finding was obtained in the hOBs after the ADSC-EV treatment (Figure 4B). In the hOBs, enhanced cell proliferation was detected in the EV group relative to the control group at the concentrations of 10^7^, 10^8^, and 10^9^ particles/mL (Figure 4). These results revealed that the ADSC-EVs did not influence the survival of osteoblasts and actually enhanced their proliferation.

### 3.4. ADSC-EVs Promote Osteoblast Activity

**Figure 5:**
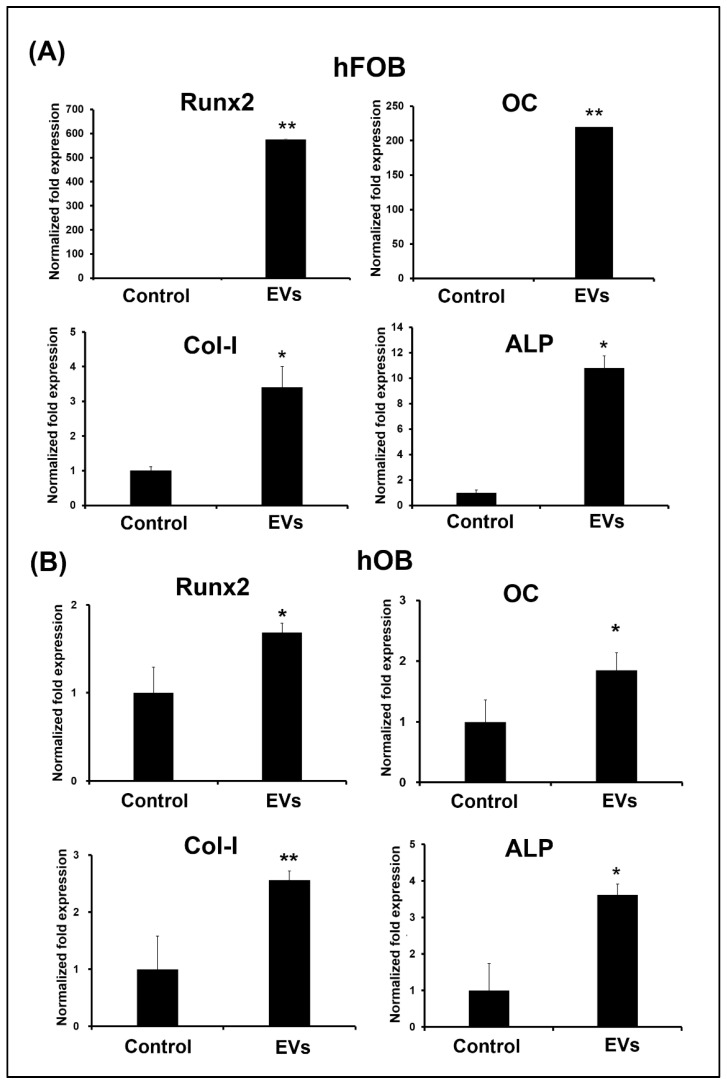
ADSC-EVs promoted osteogenic marker gene expression in osteoblasts. The hFOBs (**A**) and hOBs (**B**) were treated with ADSC-EVs at concentrations of 0 (Control: control group) or 10^9^ particles/mL (EVs: EV group) for 5 days. The mRNA expression levels of the osteogenic marker genes (runt-related transcription factor 2 (*Runx2*), osteocalcin (*OC*), collagen type I (*Col-I*), and alkaline phosphatase (*ALP*)) of hFOBs and hOBs were measured. Gene expression levels are expressed relative to the control group, which is defined as 1. Data are presented as the mean ± SD (*n* = 3). * *p* < 0.05 and ** *p* < 0.01 for comparisons with the control group.

**Figure 6:**
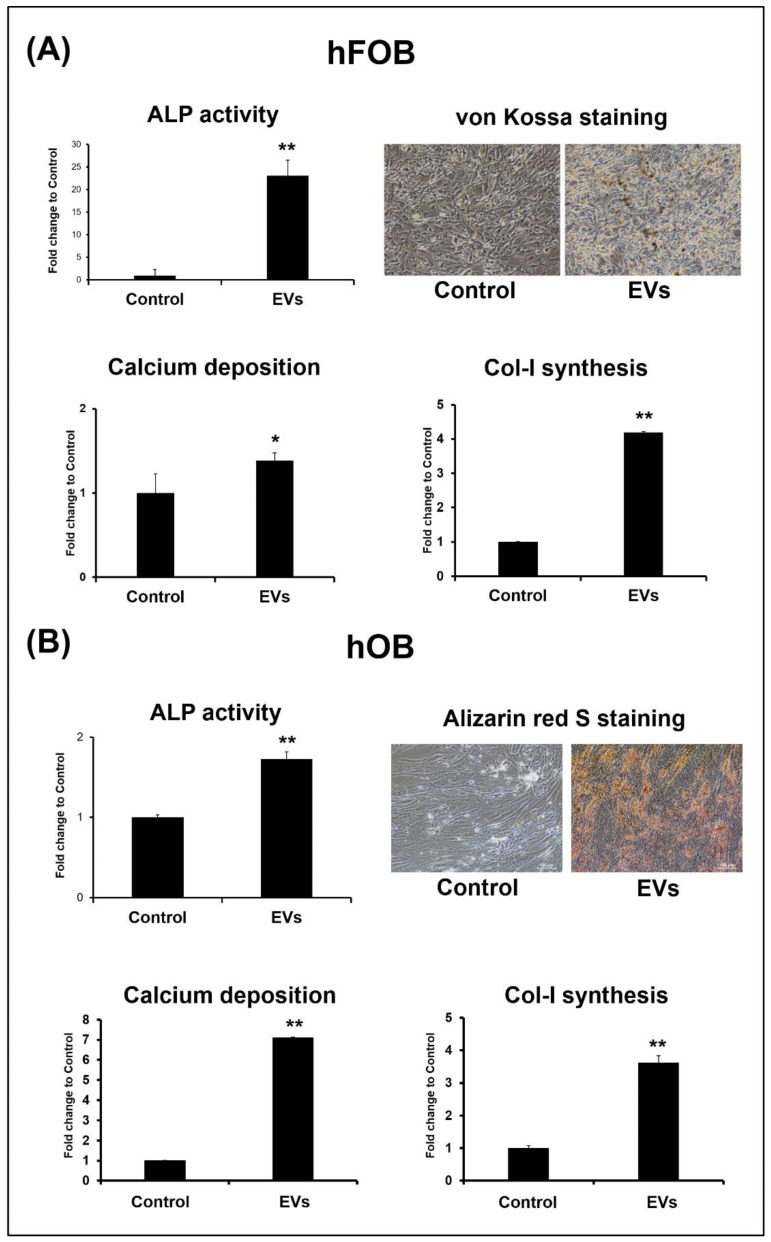
ADSC-EVs promote *ALP* activity, calcium deposition, and collagen type I (*Col-I*) synthesis in osteoblasts. The hFOBs (**A**) and hOBs (**B**) were treated with ADSC-EVs at concentrations of 0 (Control: control group) or 1 × 10^9^ particles/mL (EVs: EV group) for 12 days and analyzed through von Kossa staining, Alizarin red S staining and quantification, and ELISA for *ALP* activity and *Col-I* synthesis. Data are presented as the mean ± SD (*n* = 3). * *p* < 0.05 and ** *p* < 0.01 for comparisons with the control group.

To investigate the effect of the ADSC-EVs on osteoblast activity, the hFOBs and hOBs were treated with ADSC-EVs at a concentration of 10^9^ particles/mL for 12 days, and the mRNA expression of the osteogenic genes (*Runx2, OC, ALP,* and *Col-I*) and *ALP* activity by the hFOBs and hOBs was evaluated on day 5. The results revealed that the ADSC-EV treatment enhanced the osteogenic gene expression and *ALP* activity of the hFOBs and hOBs. In both the hFOBs and hOBs, the mRNA expression levels of *Runx2, OC, ALP*, and *Col-I* were higher in the EV group than in the control group on day 5 (Figure 5A,B, respectively). The same results were also found in *ALP* activity on day 5 (Figure 6A,B). Since we found that ADSC-EVs enhance the mRNA expression of osteogenic genes and *ALP* activity by the hFOBs and hOBs on day 5, we further confirmed the occurrence of the matrix synthesis by these cells on day 12.

To confirm the occurrence of matrix synthesis by the hFOBs and hOBs after ADSC-EV treatment, the hFOBs and hOBs were analyzed for calcium deposition and *Col-I* synthesis on day 12 after the ADSC-EV treatment. The results indicated that the calcium deposition and *Col-I* synthesis by the hFOBs and hOBs were enhanced after ADSC-EV treatment (Figure 6A,B). During von Kossa staining, there were more brown-stained areas in the extracellular matrix of the EV group of hFOBs relative to the control group of hFOBs (Figure 6A). The quantitative analyses also indicated that the calcium deposition and *Col-I* synthesis in the EV group were enhanced relative to those in the control group (Figure 6A). Similar results were obtained for the hOBs. The Alizarin red S staining results revealed more red-stained areas in the extracellular matrix of the EV group than in that of the control group (Figure 6B). The calcium deposition and *Col-I* synthesis in the EV group were enhanced relative to those in the control group (Figure 6B). Overall, the results revealed that the ADSC-EV treatment promoted osteoblast activity in vitro.

### 3.5. miRNA Bioinformatics Analysis of ADSC-EVs

**Figure 7:**
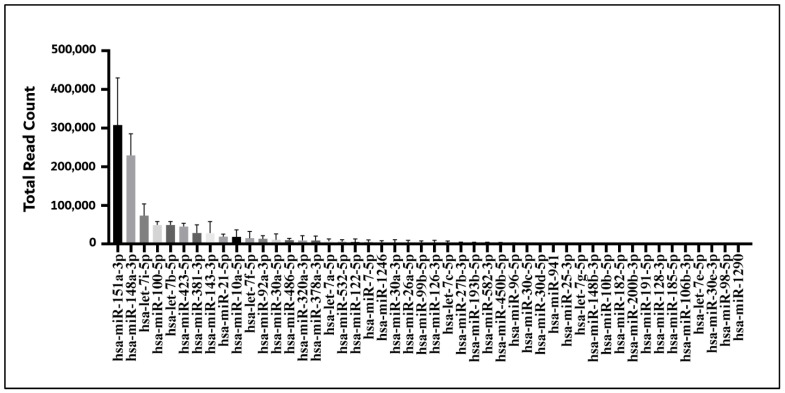
miRNA bioinformatics analysis of ADSC-EVs. Next-generation sequencing of the 48 most abundant known miRNAs detected in ADSC-EVs; the total read count is shown (*n* = 3).

**Figure 8:**
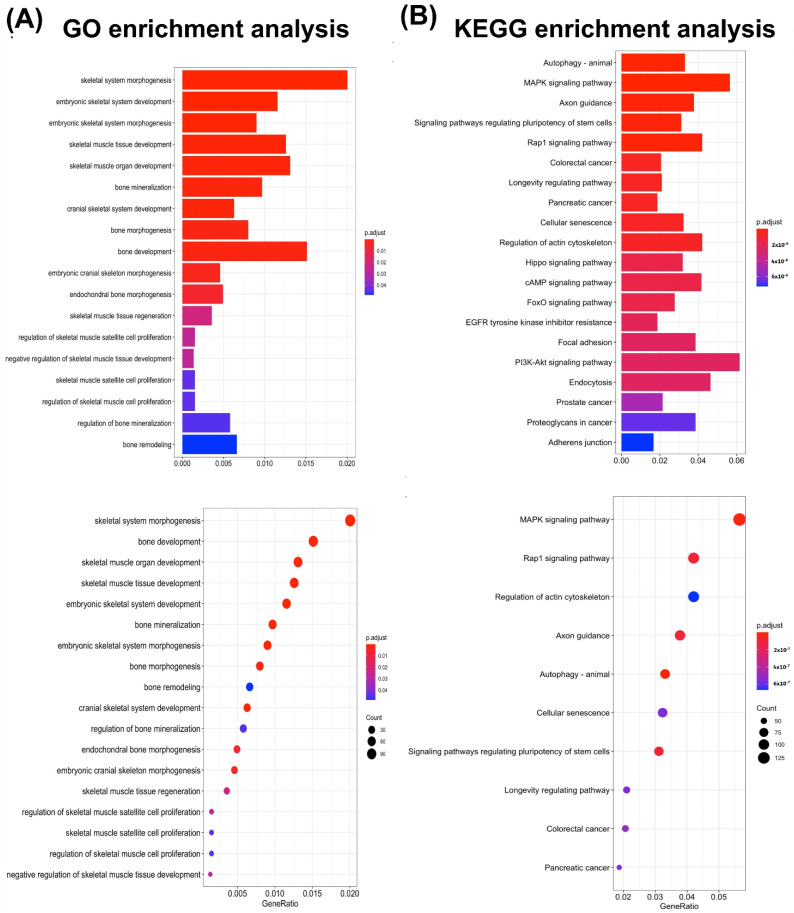
Gene ontology (GO) enrichment and Kyoto Encyclopedia of Genes and Genomes (KEGG) pathway analysis. GO pathway and KEGG pathway analyses were performed for the target genes of miRNAs enriched in ADSC-EVs. (**A**) GO enrichment analysis results. (**B**) KEGG enrichment analysis results. Bar and dot plots of GO and KEGG are shown (*n* = 3).

To analyze the miRNA profile of the ADSC-EVs, three separate batches of ADSC-EVs were isolated. The total RNA was purified from the ADSC-EVs and used for small-RNA sequencing. The 48 most abundant known miRNAs detected in the ADSC-EVs were ordered on the basis of the total read count (Figure 7); *hsa-miRNA-151a-3P*, *hsa-miRNA-148a-3P*, and *hsa-let-7i-5P* were the three most abundant miRNAs in the ADSC-EVs (Figure 7). To further investigate the possible mechanisms underlying the effects of miRNAs on osteoblast functions, these 48 miRNAs were subjected to GO and KEGG analyses to identify the pathways that were controlled by the ADSC-EVs. The GO analysis indicated that skeletal system morphogenesis and bone development were enriched pathways (Figure 8A), whereas the KEGG analysis revealed the key roles of the mitogen-activated protein kinase (*MAPK*) signaling pathway and Ras-related protein 1 (*Rap-1*) signaling pathway (Figure 8B). These results highlighted the possible contributions of the miRNAs in ADSC-EVs in promoting the osteoblast functions.

## 4. Discussion

### 4.1. ADSC-EVs Enhance Osteoblast Function and May Contribute to Bone Regeneration in ADSC-Based BTE

Osteoblasts are the main cells responsible for new bone formation [22]. Bone fracture healing in 5–10% of patients can be delayed or fail due to aging and disease (e.g., osteoporosis), and the reduced bone repair ability of osteoblasts was indicated [3,53]. For BTE, studies have demonstrated that EVs derived from MSCs are beneficial for fracture healing [3]. The body of evidence is increasingly suggesting that MSCs secrete a wide range of trophic factors to modulate various regenerative processes, including cell survival, cell proliferation, cell differentiation, and matrix synthesis [54,55]. In ADSC-based BTE, whether ADSC-EVs promote osteoblast functions remains rarely investigated. In the present study, we investigated the effect of ADSC-EVs on osteoblast functions and demonstrated that ADSC-EVs do not influence cell survival but increase osteoblast proliferation and activity in vitro (Figure 3, Figure 4, Figure 5 and Figure 6). The GO analysis revealed that the miRNAs within ADSC-EVs are highly relevant to skeletal system morphogenesis and bone development (Figure 7 and Figure 8). Moreover, the KEGG analysis indicated that these miRNAs may regulate the functions of osteoblasts through autophagy, the *MAPK* signaling pathway, or the *Rap-1* signaling pathway (Figure 7 and Figure 8). These results suggest that ADSC-EVs enhance the functions of osteoblasts and can contribute to bone regeneration in ADSC-based BTE.

### 4.2. Characteristics of Isolated ADSC-EVs Met the Minimal Information for Studies of Extracellular Vesicles (MISEV) Criteria for EVs

On the basis of their size differences, EVs are mainly divided into exosomes, microvesicles, and apoptotic bodies [56]. Distinguishing these EVs is a challenge because of their overlapping biophysical characteristics and lack of discriminating markers [57,58]. The International Society for Extracellular Vesicles (ISEV) published the Minimal Information for Studies of Extracellular Vesicles in 2014 (MISEV 2014), which is a set of guidelines for the characterization of EVs [59], and it updated these guidelines in 2018 (MISEV 2018) [39]. In the present study, ADSC-EVs were isolated and characterized per MISEV 2014 and 2018. Ultracentrifugation is a high-recovery, low-specificity method that is suitable for separating and concentrating whole EVs in a CM [39,59]; therefore, we performed ultracentrifugation to isolate whole ADSC-EVs from our CMs. Per MISEV 2014 and 2018, we characterized the ADSC-EVs in terms of size, number, morphology, and protein composition after whole ADSC-EVs were isolated [39,59]. The NTA technique is a method recommended by MISEV 2014 and 2018 for analyzing the size and number of ADSC-EVs [39,59]. Exosomes range in size from 30 to 100 nm [3], whereas the larger microvesicles range in size from 100 to 1000 nm [3]. Through NTA, we discovered that the sizes of the examined ADSC-EVs ranged from 0 to 1000 nm (Figure 1A,B), corresponding to the sizes of exosomes and microvesicles. The ISEV uses EV as a generic term for particles that are naturally released from a cell and that are delimited by a lipid bilayer and cannot replicate (i.e., lacking a functional nucleus) [39]. TEM revealed that the morphology of ADSC-EVs were spherical particles (Figure 1C). For analysis of the protein composition of EVs, the ISEV recommends that at least three positive protein markers of EVs should be tested, including at least one transmembrane/lipid-bound protein (*CD9*, *CD63*, or *CD81*) and one cytosolic protein (*Alix*) [39,59]. Furthermore, at least one negative protein marker (e.g., β-*actin*) should be tested [39,59]. In the present study, the isolated ADSC-EVs were positive for two transmembrane/lipid-bound proteins (*CD9* and *CD81*) and one cytosolic protein (*Alix*) (Figure 1D). We also discovered that cellular proteins such as actin were not present in the ADSCs [60]. β-*actin* was not detected in our isolated ADSC-EVs (Figure 1D). Collectively, the aforementioned results revealed that the ADSC-EVs were successfully isolated and that these isolated ADSC-EVs met the MISEV 2014 and 2018 criteria for EVs.

### 4.3. In ADSC-Based BTE, Cell-to-Cell Communication between ADSCs and Osteoblasts May Occur through the Delivery of ADSC-EVs to Osteoblasts

Exosomes and microvesicles are two key types of EVs used in tissue engineering [3]. Studies have demonstrated that EVs are instrumental in cell-to-cell communication [61,62]. EVs can act directly bind to specific cells [63]; they also act as paracrine signaling factors that can alter the functions of a neighboring or distant target cell [64]. To test the effect of ADSC-EVs on osteoblasts, we first investigated the uptake of the ADSC-EVs by osteoblasts and the subsequent cellular responses. Through coculturing of the ADSC-EVs with hFOBs and hOBs, we discovered that the uptake of the ADSC-EVs by the hFOBs and hOBs occurred on day 5 (Figure 2). These results suggest that the cell-to-cell communication between ADSCs and osteoblasts occurs through the delivery of ADSC-EVs to osteoblasts in ADSC-based BTE.

### 4.4. ADSC-EVs May Enhance Osteoblast Proliferation in ADSC-Based BTE

Increasing osteoblast proliferation can enhance bone formation [65,66]. However, the source of osteoblasts is limited in vivo [53]. Moreover, a reduction in the number of osteoblasts caused by decreased proliferation or apoptosis is indicative of aging or osteoporosis [53]. In the present study, we tested whether ADSC-EV treatment affects the survival and proliferation of osteoblasts, and we discovered that this treatment did not change cell survival but enhanced the proliferation of hFOBs and hOBs (Figure 3 and Figure 4). These results suggest that ADSC-EVs enhance osteoblast proliferation during bone regeneration in ADSC-based BTE.

### 4.5. ADSC-EVs May Enhance Osteoblast Activity in ADSC-Based BTE

Osteoblast activity is essential to the extracellular matrix synthesis of bone [22,67]. Osteoblast activity is the highest during bone formation and growth [68]. When regeneration is required to fix a bone defect, osteoblasts are activated [68,69]. However, aging or osteoporosis cause the osteoblast activity to decrease [53]. Bone consists of osteocytes that are derived from the osteoblasts embedded in a mineralized extracellular matrix, which has inorganic and organic components [22]. The inorganic component is primarily calcium [22], and the organic component is primarily *Col-I* (>90%) [22]. Calcium is deposited in the form of hydroxyapatite, and *Col-I* provides the structural support for bones [22]. Osteoblasts secrete bone matrix proteins, including *Col-I*, *OC*, and *ALP* [68,69]. The expression of *Runx2* marks the commitment to a mature osteoblast [22]. Mature osteoblasts secrete *OC*, *ALP*, and *Col-I*, which are the predominant components of the matrix; thereafter, hydroxyapatite is formed through calcium phosphate mineralization [53]. To determine whether ADSC-EVs enhance osteoblast activity, we analyzed the osteoblasts’ mRNA expressions of osteogenic genes (*Runx2*, *ALP*, *OC*, and *Col-I*) and ALP activity. We also tested the bone extracellular matrix synthesis (calcium and *Col-I*) of the osteoblasts after the ADSC-EV treatment, and we discovered that the ADSC-EV treatment increased the mRNA expression levels of the osteogenic genes (*Runx2*, *ALP*, *OC*, and *Col-I*) and *ALP* activity by hFOBs and hOBs on day 5 (Figure 5 and Figure 6). The extracellular matrix related to bone synthesis (calcium deposition and *Col-I*) achieved by the hFOBs and hOBs was also increased after the ADSC-EV treatment (Figure 6). Overall, these results suggest that ADSC-EVs enhance the osteoblast activity during bone regeneration in ADSC-based BTE.

### 4.6. ADSC-EVs Enhance Osteoblast Function and That Their miRNAs May Contribute to the Effect

EVs are regarded as the regulators of cell-to-cell communication, which is achieved through the transfer of lipids, nucleic acids (mRNAs and miRNAs), and cell-specific proteins to recipient cells to elicit the cellular responses of the recipient cells [61,62]. miRNAs are a class of small, noncoding RNA molecules that function as negative regulators of gene expression at the posttranscriptional level [70]. EVs can modulate recipient cells through the miRNA regulation of posttranscriptional coding genes [71]. Therefore, we analyzed and profiled the miRNAs of ADSC-EVs on the basis of the total read count. The three most abundant miRNAs in the ADSC-EVs were discovered to be *hsa-miR-151a-3p*, *hsa-miR-148a-3p*, and *hsa-let-7i-5p* (Figure 7). Among these three miRNAs, *miR-151a-3p* and *miR-148a-3p* were reported to regulate osteoblast activity [72,73], and *let-7i-5p* was shown to promote the osteogenic differentiation of MSCs [74]. These results suggest that ADSC-EVs enhance osteoblast functions and that their miRNA may contribute to the effect.

### 4.7. The miRNAs in ADSC-EVs Enhance Osteoblast Function May through Factors, including Autophagy, the MAPK Signaling Pathway, and the Rap-1 Signaling Pathway

We performed GO pathway and KEGG pathway analyses for miRNAs enriched in ADSC-EVs to clarify their possible molecular mechanism. The GO analysis indicated that skeletal system morphogenesis and bone development were enriched pathways (Figure 8A). The KEGG pathway analysis revealed that autophagy, the *MAPK* signaling pathway, and the *Rap-1* signaling pathway may make contributions (Figure 8B). Osteoblast autophagy is involved in mineralization and bone homeostasis [75]. *MAPK* was revealed to play a key role in skeletal development and bone homeostasis, which particularly affect osteoblast differentiation [76]. Of the three classic *MAPKs*, the *p38* and *ERK* activity levels are crucial in determining and shaping the skeleton [76]. The *Rap-1* pathway may also make a contribution; one study reported that *RAP-1A* is a key regulator in osteoblast differentiation because of the phosphorylation of *ERK* and *P38* expression [77]. Among the three most abundant miRNAs detected in the ADSC-EVs, *miR-151* regulates the autophagy process [78], *miR-148a-3p* was demonstrated to be involved in osteoblast differentiation [79], and miRNA *let-7i-5p* was reported to be a positive regulator of bone development [80]. Our results suggested that ADSC-EVs influence osteoblast functions through factors including autophagy, the *MAPK* signaling pathway, and the *Rap-1* signaling pathway. Although we showed that ADSC-EVs enhance the osteoblast functions and that their miRNAs may contribute to the effects, whether these miRNAs influence osteoblastic functions through autophagy, *MAPK* signaling, or *Rap-1* signaling has not yet been fully clarified. Further in-depth investigations are necessary regarding the molecular mechanism of the miRNAs carried by ADSC-EVs in relation to osteoblastic functions.

### 4.8. The Difference in Amount of mRNA Expressions in Rnux2 and OC between hFOB and hOB after ADSC-EV Treatments May Be Due to Runx2 Phosphorylation

Here, we found that *Runx2* and *OC* expressions were increased 600 and 200 times in hFOBs after ADSC-EV treatments (Figure 5A). However, *Runx2* and *OC* only increased two times in hOBs (Figure 5B). *Runx2* phosphorylation is important in the regulation of *Runx2* activity and the osteogenesis process [81]. It has been reported that the stability of *Runx2* is maintained by *PEBP2β*, and it heterodimerized with *Runx2* and, thus, regulates the stability of *Runx2* [81]. Studies have shown that the phosphorylation of *Runx2* at the serine residues S104 and S451 negatively regulated the heterodimerization [81]. Moreover, *Rnux2* is known to regulate the expression of *OC* [81]. The phosphorylation of *Runx2* leads to either a positive or a negative regulation of its target gene expression [81]. Overall, the difference in the amount of mRNA expressions in *Rnux2* and *OC* between hFOBs and hOBs after ADSC-EV treatments may be due to *Runx2* phosphorylation. However, further investigations are required.

## 5. Conclusions

In conclusion, we isolated and characterized ADSC-EVs and demonstrated that they mediate the function of osteoblasts by enhancing osteoblast proliferation and activity. Our study suggests that the effect of ADSC-EVs on osteoblast functions contributes to bone regeneration during ADSC-based BTE.

## Data Availability

Not applicable.

## References

[1] Ho-Shui-Ling A., Bolansder J., Rustom L.E., Johnson A.W., Luyten F.P., Picart C. (2018). Bone regeneration strategies: Engineered scaffolds, bioactive molecules and stem cells current stage and future perspectives. Biomaterials.

[2] Einhorn T.A., Gerstenfeld L.C. (2015). Fracture healing: Mechanisms and interventions. Nat. Rev. Rheumatol..

[3] Mende W., Gotzl R., Kubo Y., Pufe T., Ruhl T., Beier J.P. (2021). The Role of Adipose Stem Cells in Bone Regeneration and Bone Tissue Engineering. Cells.

[4] Barba M., Di Taranto G., Lattanzi W. (2017). Adipose-derived stem cell therapies for bone regeneration. Expert Opin. Biol. Ther..

[5] Gomez-Barrena E., Rosset P., Lozano D., Stanovici J., Ermthaller C., Gerbhard F. (2015). Bone fracture healing: Cell therapy in delayed unions and nonunions. Bone.

[6] Amini A.R., Laurencin C.T., Nukavarapu S.P. (2012). Bone tissue engineering: Recent advances and challenges. Crit. Rev. Biomed. Eng..

[7] Storti G., Scioli M.G., Kim B.S., Orlandi A., Cervelli V. (2019). Adipose-Derived Stem Cells in Bone Tissue Engineering: Useful Tools with New Applications. Stem Cells Int..

[8] Marolt Presen D., Traweger A., Gimona M., Redl H. (2019). Mesenchymal Stromal Cell-Based Bone Regeneration Therapies: From Cell Transplantation and Tissue Engineering to Therapeutic Secretomes and Extracellular Vesicles. Front. Bioeng. Biotechnol..

[9] Nancarrow-Lei R., Mafi P., Mafi R., Khan W. (2017). A Systemic Review of Adult Mesenchymal Stem Cell Sources and their Multilineage Differentiation Potential Relevant to Musculoskeletal Tissue Repair and Regeneration. Curr. Stem Cell Res. Ther..

[10] Al-Ghadban S., Artiles M., Bunnell B.A. (2021). Adipose Stem Cells in Regenerative Medicine: Looking Forward. Front. Bioeng. Biotechnol..

[11] Marie P.J. (2013). Targeting integrins to promote bone formation and repair. Nat. Rev. Endocrinol..

[12] Tan S.H.S., Wong J.R.Y., Sim S.J.Y., Tjio C.K.E., Wong K.L., Chew J.R.J., Hui J.H.P., Toh W.S. (2020). Mesenchymal stem cell exosomes in bone regenerative strategies-a systematic review of preclinical studies. Mater. Today Bio..

[13] Prockop D.J., Gregory C.A., Spees J.L. (2003). One strategy for cell and gene therapy: Harnessing the power of adult stem cells to repair tissues. Proc. Natl. Acad. Sci. USA.

[14] De Jong O.G., Van Balkom B.W., Schiffelers R.M., Bouten C.V., Verhaar M.C. (2014). Extracellular vesicles: Potential roles in regenerative medicine. Front. Immunol..

[15] Tieu A., Lalu M.M., Slobodian M., Gnyra C., Fergusson D.A., Montroy J., Burger D., Stewart D.J., Allan D.S. (2020). An Analysis of Mesenchymal Stem Cell-Derived Extracellular Vesicles for Preclinical Use. ACS Nano.

[16] Xu L., Gao S., Zhou R., Zhou F., Qiao Y., Qiu D. (2020). Bioactive Pore-Forming Bone Adhesives Facilitating Cell Ingrowth for Fracture Healing. Adv. Mater..

[17] Salgado A.J., Reis R.L., Sousa N.J., Gimble J.M. (2010). Adipose tissue derived stem cells secretome: Soluble factors and their roles in regenerative medicine. Curr. Stem Cell Res. Ther..

[18] Van der Pol E., Boing A.N., Harrison P., Sturk A., Nieuwland R. (2012). Classification, functions, and clinical relevance of extracellular vesicles. Pharmacol. Rev..

[19] Turturici G., Tinnirello R., Sconzo G., Geraci F. (2014). Extracellular membrane vesicles as a mechanism of cell-to-cell communication: Advantages and disadvantages. Am. J. Physiol. Cell Physiol..

[20] Li W., Liu Y., Zhang P., Tang Y., Zhou M., Jiang W., Zhang X., Wu G., Zhou Y. (2018). Tissue-Engineered Bone Immobilized with *Human* Adipose Stem Cells-Derived Exosomes Promotes Bone Regeneration. ACS Appl. Mater. Interfaces.

[21] Mou S., Zhou M., Li Y., Wang J., Yuan Q., Xiao P., Sun J., Wang Z. (2019). Extracellular Vesicles from *Human* Adipose-Derived Stem Cells for the Improvement of Angiogenesis and Fat-Grafting Application. Plast. Reconstr. Surg..

[22] Salhotra A., Shah H.N., Levi B., Longaker M.T. (2020). Mechanisms of bone development and repair. Nat. Rev. Mol. Cell Biol..

[23] Makino T., Tsukazaki H., Ukon Y., Tateiwa D., Yoshikawa H., Kaito T. (2018). The Biological Enhancement of Spinal Fusion for Spinal Degenerative Disease. Int. J. Mol. Sci..

[24] Henry J.P., Bordoni B. (2021). Histology, Osteoblasts.

[25] Kito H., Ohya S. (2021). Role of K^+^ and Ca^2+^-Permeable Channels in Osteoblast Functions. Int. J. Mol. Sci..

[26] Shao Y.Z., Chen S., Zhou Y.S. (2020). Applications of stem cell-derived extracellular vesicles in bone regenerative therapy. Zhonghua Kou Qiang Yi Xue Za Zhi.

[27] Chen S., Tang Y., Liu Y., Zhang P., Lv L., Zhang X., Jia L., Zhou Y. (2019). Exosomes derived from miR-375-overexpressing *human* adipose mesenchymal stem cells promote bone regeneration. Cell Prolif..

[28] Tofino-Vian M., Guillen M.I., Perez Del Caz M.D., Castejon M.A., Alcaraz M.J. (2017). Extracellular Vesicles from Adipose-Derived Mesenchymal Stem Cells Downregulate Senescence Features in Osteoarthritic Osteoblasts. Oxid. Med. Cell Longev..

[29] Yang X., Yang J., Lei P., Wen T. (2019). LncRNA MALAT1 shuttled by bone marrow-derived mesenchymal stem cells-secreted exosomes alleviates osteoporosis through mediating microRNA-34c/SATB2 axis. Aging.

[30] Zhang Y., Cao X., Li P., Fan Y., Zhang L., Ma X., Sun R., Liu Y., Li W. (2021). microRNA-935-modified bone marrow mesenchymal stem cells-derived exosomes enhance osteoblast proliferation and differentiation in osteoporotic rats. Life Sci..

[31] Chen H.T., Lee M.J., Chen C.H., Chuang S.C., Chang L.F., Ho M.L., Hung S.H., Fu Y.C., Wang Y.H., Wang H.I. (2012). Proliferation and differentiation potential of *human* adipose-derived mesenchymal stem cells isolated from elderly patients with osteoporotic fractures. J. Cell Mol. Med..

[32] Wu S.C., Chen C.H., Wang J.Y., Lin Y.S., Chang J.K., Ho M.L. (2018). Hyaluronan size alters chondrogenesis of adipose-derived stem cells via the CD44/ERK/SOX-9 pathway. Acta Biomater..

[33] Nemcakova I., Litvinec A., Mandys V., Potocky S., Plencner M., Doubkova M., Nanka O., Olejnickova V., Sankova B., Bartos M. (2022). Coating Ti6Al4V implants with nanocrystalline diamond functionalized with BMP-7 promotes extracellular matrix mineralization in vitro and faster osseointegration in vivo. Sci. Rep..

[34] Struber A., Auer G., Fischlechner M., Wickstrom C., Reiter L., Lutsch E., Simon-Nobbe B., Marozin S., Lepperdinger G. (2022). Low-Cost Devices for Three-Dimensional Cell Aggregation, Real-Time Monitoring Microscopy, Microfluidic Immunostaining, and Deconvolution Analysis. Bioengineering.

[35] Li C.J., Chang J.K., Chou C.H., Wang G.J., Ho M.L. (2010). The PI3K/Akt/FOXO3a/p27Kip1 signaling contributes to anti-inflammatory drug-suppressed proliferation of *human* osteoblasts. Biochem. Pharmacol..

[36] Purushothaman A. (2019). Exosomes from Cell Culture-Conditioned Medium: Isolation by Ultracentrifugation and Characterization. Methods Mol. Biol..

[37] Eirin A., Riester S.M., Zhu X.Y., Tang H., Evans J.M., O’Brien D., van Wijnen A.J., Lerman L.O. (2014). MicroRNA and mRNA cargo of extracellular vesicles from porcine adipose tissue-derived mesenchymal stem cells. Gene.

[38] Derfus B.A., Rachow J.W., Mandel N.S., Boskey A.L., Buday M., Kushnaryov V.M., Ryan L.M. (1992). Articular cartilage vesicles generate calcium pyrophosphate dihydrate-like crystals in vitro. Arthritis Rheum..

[39] Théry C., Witwer K.W., Aikawa E., Alcaraz M.J., Anderson J.D., Andriantsitohaina R., Antoniou A., Arab T., Archer F., Atkin-Smith G.K. (2018). Minimal information for studies of extracellular vesicles 2018 (MISEV2018): A position statement of the International Society for Extracellular Vesicles and update of the MISEV2014 guidelines. J. Extracell. Vesicles.

[40] Wu S.C., Huang P.Y., Chen C.H., Teong B., Chen J.W., Wu C.W., Chang J.K., Ho M.L. (2018). Hyaluronan microenvironment enhances cartilage regeneration of *human* adipose-derived stem cells in a chondral defect model. Int. J. Biol. Macromol..

[41] Relic B., Guicheux J., Mezin F., Lubberts E., Togninalli D., Garcia I., van den Berg W.B., Guerne P.A. (2001). Il-4 and IL-13, but not IL-10, protect *human* synoviocytes from apoptosis. J. Immunol..

[42] Ma W.J., Ruys A.J., Mason R.S., Martin P.J., Bendavid A., Liu Z., Ionescu M., Zreiqat H. (2007). DLC coatings: Effects of physical and chemical properties on biological response. Biomaterials.

[43] Magne D., Bluteau G., Faucheux C., Palmer G., Vignes-Colombeix C., Pilet P., Rouillon T., Caverzasio J., Weiss P., Daculsi G. (2003). Phosphate is a specific signal for ATDC5 chondrocyte maturation and apoptosis-associated mineralization: Possible implication of apoptosis in the regulation of endochondral ossification. J. Bone Miner. Res..

[44] Livak K.J., Schmittgen T.D. (2001). Analysis of relative gene expression data using real-time quantitative PCR and the 2(-Delta Delta C(T)) Method. Methods.

[45] Lin T.M., Tsai J.L., Lin S.D., Lai C.S., Chang C.C. (2005). Accelerated growth and prolonged lifespan of adipose tissue-derived *human* mesenchymal stem cells in a medium using reduced calcium and antioxidants. Stem Cells Dev..

[46] Takacs R., Matta C., Somogyi C., Juhasz T., Zakany R. (2013). Comparative analysis of osteogenic/chondrogenic differentiation potential in primary limb bud-derived and C3H10T1/2 cell line-based mouse micromass cultures. Int. J. Mol. Sci..

[47] Liu X., Yan Z., Wu C., Yang Y., Li X., Zhang G. (2019). FastProNGS: Fast preprocessing of next-generation sequencing reads. BMC Bioinform..

[48] Blankenberg D., Gordon A., Von Kuster G., Coraor N., Taylor J., Nekrutenko A., Galaxy T. (2010). Manipulation of FASTQ data with Galaxy. Bioinformatics.

[49] Li H., Durbin R. (2009). Fast and accurate short read alignment with Burrows-Wheeler transform. Bioinformatics.

[50] Robinson M.D., McCarthy D.J., Smyth G.K. (2010). edgeR: A Bioconductor package for differential expression analysis of digital gene expression data. Bioinformatics.

[51] Yu G., Wang L.G., Han Y., He Q.Y. (2012). clusterProfiler: An R package for comparing biological themes among gene clusters. Omics.

[52] Wickham H. (2016). ggplot2: Elegant Graphics for Data Analysis.

[53] Pignolo R.J., Law S.F., Chandra A. (2021). Bone Aging, Cellular Senescence, and Osteoporosis. JBMR Plus.

[54] Meirelles Lda S., Fontes A.M., Covas D.T., Caplan A.I. (2009). Mechanisms involved in the therapeutic properties of mesenchymal stem cells. Cytokine Growth Factor Rev..

[55] Toh W.S., Foldager C.B., Pei M., Hui J.H. (2014). Advances in mesenchymal stem cell-based strategies for cartilage repair and regeneration. Stem Cell Rev..

[56] He C., Zheng S., Luo Y., Wang B. (2018). Exosome Theranostics: Biology and Translational Medicine. Theranostics.

[57] Van Dommelen S.M., Vader P., Lakhal S., Kooijmans S.A., van Solinge W.W., Wood M.J., Schiffelers R.M. (2012). Microvesicles and exosomes: Opportunities for cell-derived membrane vesicles in drug delivery. J. Control Release.

[58] Cocucci E., Racchetti G., Meldolesi J. (2009). Shedding microvesicles: Artefacts no more. Trends Cell Biol..

[59] Lotvall J., Hill A.F., Hochberg F., Buzas E.I., Di Vizio D., Gardiner C., Gho Y.S., Kurochkin I.V., Mathivanan S., Quesenberry P. (2014). Minimal experimental requirements for definition of extracellular vesicles and their functions: A position statement from the International Society for Extracellular Vesicles. J. Extracell. Vesicles.

[60] Katsuda T., Tsuchiya R., Kosaka N., Yoshioka Y., Takagaki K., Oki K., Takeshita F., Sakai Y., Kuroda M., Ochiya T. (2013). *Human* adipose tissue-derived mesenchymal stem cells secrete functional neprilysin-bound exosomes. Sci. Rep..

[61] Gyorgy B., Szabo T.G., Pasztoi M., Pal Z., Misjak P., Aradi B., Laszlo V., Pallinger E., Pap E., Kittel A. (2011). Membrane vesicles, current state-of-the-art: Emerging role of extracellular vesicles. Cell. Mol. Life Sci..

[62] Camussi G., Deregibus M.C., Bruno S., Cantaluppi V., Biancone L. (2010). Exosomes/microvesicles as a mechanism of cell-to-cell communication. Kidney Int..

[63] Losche W., Scholz T., Temmler U., Oberle V., Claus R.A. (2004). Platelet-derived microvesicles transfer tissue factor to monocytes but not to neutrophils. Platelets.

[64] Van Niel G., D’Angelo G., Raposo G. (2018). Shedding light on the cell biology of extracellular vesicles. Nat. Rev. Mol. Cell Biol..

[65] Ducy P., Schinke T., Karsenty G. (2000). The osteoblast: A sophisticated fibroblast under central surveillance. Science.

[66] Lane N.E., Kelman A. (2003). A review of anabolic therapies for osteoporosis. Arthritis Res. Ther..

[67] Ottewell P.D. (2016). The role of osteoblasts in bone metastasis. J. Bone Oncol.

[68] Rutkovskiy A., Stenslokken K.O., Vaage I.J. (2016). Osteoblast Differentiation at a Glance. Med. Sci. Monit. Basic Res..

[69] Jensen E.D., Gopalakrishnan R., Westendorf J.J. (2010). Regulation of gene expression in osteoblasts. Biofactors.

[70] Ambros V. (2004). The functions of animal microRNAs. Nature.

[71] Valadi H., Ekstrom K., Bossios A., Sjostrand M., Lee J.J., Lotvall J.O. (2007). Exosome-mediated transfer of mRNAs and microRNAs is a novel mechanism of genetic exchange between cells. Nat. Cell Biol..

[72] Wang Y., Zhang H., Yang G., Xiao L., Li J., Guo C. (2020). Dysregulated Bone Metabolism Is Related to High Expression of *miR-151a-3p* in Severe Adolescent Idiopathic Scoliosis. BioMed Res. Int..

[73] Liu N., Sun Y. (2021). *microRNA-148a-3p*-targeting p300 protects against osteoblast differentiation and osteoporotic bone reconstruction. Regen. Med..

[74] Zhang Y., Cheng W., Han B., Guo Y., Wei S., Yu L., Zhang X. (2021). *Let-7i-5p* functions as a putative osteogenic differentiation promoter by targeting CKIP-1. Cytotechnology.

[75] Nollet M., Santucci-Darmanin S., Breuil V., Al-Sahlanee R., Cros C., Topi M., Momier D., Samson M., Pagnotta S., Cailleteau L. (2014). Autophagy in osteoblasts is involved in mineralization and bone homeostasis. Autophagy.

[76] Rodriguez-Carballo E., Gamez B., Ventura F. (2016). *p38 MAPK* Signaling in Osteoblast Differentiation. Front. Cell Dev. Biol..

[77] Wu Y., Zhou J., Li Y., Zhou Y., Cui Y., Yang G., Hong Y. (2015). *Rap1A* Regulates Osteoblastic Differentiation via the *ERK* and *p38* Mediated Signaling. PLoS ONE.

[78] Liang Q., Dong W., Ou M., Li Z., Liu C., Wang F., Liu Y., Wang W. (2021). *miR-151* Affects Low-Temperature Tolerance of Penaeus vannamei by Modulating Autophagy Under Low-Temperature Stress. Front. Cell Dev. Biol..

[79] Yuan H., Xu X., Feng X., Zhu E., Zhou J., Wang G., Tian L., Wang B. (2019). A novel long noncoding RNA *PGC1beta-OT1* regulates adipocyte and osteoblast differentiation through antagonizing miR-148a-3p. Cell Death Differ..

[80] Wei J., Li H., Wang S., Li T., Fan J., Liang X., Li J., Han Q., Zhu L., Fan L. (2014). *let-7* enhances osteogenesis and bone formation while repressing adipogenesis of *human* stromal/mesenchymal stem cells by regulating HMGA2. Stem Cells Dev..

[81] Vimalraj S., Arumugam B., Miranda P.J., Selvamurugan N. (2015). *Runx2*: Structure, function, and phosphorylation in osteoblast differentiation. Int. J. Biol. Macromol..

